# Modulation of human uterine smooth muscle cell collagen contractility by thrombin, Y-27632, TNF alpha and indomethacin

**DOI:** 10.1186/1477-7827-7-2

**Published:** 2009-01-08

**Authors:** Joan Fitzgibbon, John J Morrison, Terry J Smith, Margaret O'Brien

**Affiliations:** 1 National Centre for Biomedical and Engineering Science, Orbsen Building, National University of Ireland Galway, University Road, Galway, Ireland; 2 Department of Obstetrics and Gynaecology, Clinical Science Institute, University College Hospital Galway, Newcastle Road, Galway, Ireland

## Abstract

**Background:**

Preterm labour occurs in approximately 10% of pregnancies and is a major cause of infant morbidity and mortality. However, the pathways involved in regulating contractility in normal and preterm labour are not fully elucidated. Our aim was to utilise a human myometrial contractility model to investigate the effect of a number of uterine specific contractility agents in this system. Therefore, we investigated the contractile response of human primary uterine smooth muscle cells or immortalised myometrial smooth muscle cells cultured within collagen lattices, to known mediators of uterine contractility, which included thrombin, the ROCK-1 inhibitor Y-27632, tumour necrosis factor alpha (TNF alpha) and the non-steroidal anti-inflammatory indomethacin.

**Methods:**

Cell contractility was calculated over time, with the collagen gel contraction assay, utilising human primary uterine smooth muscle cells (hUtSMCs) and immortalised myometrial smooth muscle cells (hTERT-HM): a decrease in collagen gel area equated to an increase in contractility. RNA was isolated from collagen embedded cells and gene expression changes were analysed by real time fluorescence reverse transcription polymerase chain reaction. Scanning electron and fluorescence microscopy were employed to observe cell morphology and cell collagen gel interactions. Statistical analysis was performed using ANOVA followed by Tukey's post hoc tests.

**Results:**

TNF alpha increased collagen contractility in comparison to the un-stimulated collagen embedded hUtSMC cells, which was inhibited by indomethacin, while indomethacin alone significantly inhibited contraction. Thrombin augmented the contractility of uterine smooth muscle cell and hTERT-HM collagen gels, this effect was inhibited by the thrombin specific inhibitor, hirudin. Y-27632 decreased both basal and thrombin-induced collagen contractility in the hTERT-HM embedded gels. mRNA expression of the thrombin receptor, F2R was up-regulated in hUtSMCs isolated from collagen gel lattices, following thrombin-stimulated contractility.

**Conclusion:**

TNF alpha and thrombin increased uterine smooth muscle cell collagen contractility while indomethacin had the opposite effect. Thrombin-induced collagen contractility resulted in F2R activation which may in part be mediated by the ROCK-1 pathway. This study established the in vitro human myometrial model as a viable method to assess the effects of a range of uterotonic or uterorelaxant agents on contractility, and also permits investigation of the complex regulatory pathways involved in mediating myometrial contractility at labour.

## Background

Preterm labour occurs in approximately 10% of pregnancies and is a major cause of infant morbidity and mortality. It accounts for approximately 75% of all neonatal problems. Despite major advances in obstetrics the rate of preterm births has not declined in 30 years [1,2]. To aid the decrease in the incidence of preterm labour a greater knowledge of the pathways regulating labour is necessary. An understanding of myometrial contractility at this time would therefore assist in our biological comprehension of preterm labour.

A suitable model is essential to study the processes involved in myometrial contraction and the agents which mediate these responses. The use of *in vivo *studies is not ideal as the process of labour differs amongst animals, and human parturition is distinct from other mammals, so use of animal models can only give limited insight [3]. Myometrial tissue strips have been used for tissue bath experiments, but ethical constraints and difficulties in obtaining samples limit the use of this system. Furthermore, it is often not possible to acquire enough tissue to perform adequate studies. It is also less problematic to monitor gene expression changes in this system in comparison to that in the myometrial strip sections.

Bell *et al. *described the formation of a 'tissue-like structure' produced by seeding fibroblasts in a collagen matrix and collagen lattices are now widely used as *in vitro *models for many diverse purposes [4]. Collagen is an ideal substrate as it forms the main component of the extra-cellular matrix *in vivo *and it does not cause cytotoxicity. Furthermore, preparation of lattices can be altered to best mimic the tissue of interest. Using an isolated cell system allows study of effects on cells by individual stimuli, without interference from other sources. Type 1 collagen is widely available, so studies are not restricted in sample number. With tissue strip experiments inherent differences exist between myometrial samples whereas with this system a uniform solution of cells and collagen is used, ensuring that all replicates are comparable. Other investigators in the field of myometrial reproductive biology have utilised collagen contractility assays, Dallot *et al*. monitored the effects of endothelin-1 on the contractility of smooth muscle cells derived from human myometrium, used between passages 3 and 6 [5]. Devost and Zingg determined the effect of oxytocin and its inhibitors on two human myometrial cell lines: one a telomerase-immortalised human myometrial cells (hTERT-C3), a subclone of hTERT-HM cells [6], the other a cell line derived from a primary culture of human myometrial cells [7].

Thrombin is a serine protease that converts fibrinogen to fibrin in the coagulation cascade. It also mediates cellular events through via activation of the protease activated receptors, it is proposed that F2R is the main receptor responsible for mediating these responses [8]. It had previously been demonstrated that thrombin is responsible for stimulating myometrial contractility *in vitro *and *in vivo *[9-11]. We have reported an increase in mRNA and protein expression of F2R and F2RL3 at labour [12]. Parturition is characterised by an influx of inflammatory cells into the uterus [13,14]. Moreover, several proinflammatory cytokines have been implicated in labour onset, including the pleiotropic inflammatory cytokine tumour necrosis factor α (TNFα)[15]. The non-steroidal anti-inflammatory drug indomethacin is a non-selective inhibitor of the COX 1 and 2 enzymes that catalyse the formation of prostaglandins and thromboxane from arachidonic acid. It is a tocolytic agent that can delay delivery beyond 37 weeks [16]. The small GTPase Rho and its downstream effectors, the Rho-associated coiled-coil forming protein serine/threonine kinase (ROCK) family, have been implicated in various cellular functions including vascular and smooth muscle contraction [17]. Previous work from our group has established the importance of the Rho/ROCK-1 pathway in the human myometrium at labour [18]. Y-27632 is a well characterised selective inhibitor of Rho-associated protein kinase 1.

Our aim was to exploit a human *in vitro *myometrial model to investigate the effect of known *in vivo *stimulators or relaxants of uterine contractility, with a future objective to assess unknown tocolytic compounds. A collagen contraction assay system using commercial human primary uterine smooth muscle cells (hUtSMCs) or immortalised human myometrial smooth muscle cells (hTERT-HM) was utilised to analyse the response to thrombin, Y-27632, TNFα and indomethacin. We investigated the expression of the thrombin receptor *F2R*, in the thrombin stimulated gels. Scanning electron and fluorescence microscopy methods were employed to observe cell morphology within the collagen gels.

## Methods

### Chemicals

Thrombin, hirudin, TNFα, indomethacin and rat tail collagen type I were purchased from Sigma (Dublin, Ireland). Y-27632 was from Calbiochem (San Diego, CA, USA). Stock solutions of thrombin, hirudin, TNFα and Y-27632 were dissolved in deionised water. A stock solution of 100 μM indomethacin was made in ethanol. Dulbecco's Modified Eagle Medium (DMEM), F-12 media, and foetal bovine serum (FBS) were from Invitrogen (Carlsbad, CA, USA).

### Cell culture

Human primary uterine smooth muscle cells, hUtSMCs (Cambrex, Wokingham, Berkshire, UK) were expanded in DMEM high glucose with 10% FBS (Invitrogen, USA). Myometrial human telomerase reverse transcriptase (hTERT-HM) cells kindly provided by Dr. Jennifer C. Condon [6] were cultured in DMEM-F-12/10% FBS (Invitrogen, USA). HEK293 cells were maintained in DMEM medium (Invitrogen, USA).

### Collagen contractility assay

Collagen gels were prepared from rat tail Type 1 collagen (Sigma, Ireland), to a final concentration of 1.5 mg/ml, and seeded in 24 well culture dishes, with 150,000 hUtSMC (passages 5–8), hTERT-HM or HEK293 cells per well, based on the technique described by Dallot *et al*. (4). Cells in collagen gels were allowed to equilibrate overnight in serum free DMEM medium. Gels were released from the culture dishes and various test agents of interest were added to the serum free media, inhibitors were added 30 min prior to treatment and release. The vehicle controls for thrombin, hirudin, TNFα, and Y-27632 were serum free media and for the indomethacin experiments it was 5% indomethacin, in serum free medium. Gel images were captured over time, using a FluorchemTM 8900 imager (Alpha Innotech Corporation, San Leandro, CA, USA) and the area (cm^2^) of the gels measured using Image J software . For each condition, collagen contraction was determined in quadruplicate, at a minimum. Each experiment was performed at least 3 times. Results were expressed as mean gel area (cm2) ± the standard error of the mean (SEM). 10% (vol/vol) FBS was used as a positive control for contraction in all experiments, and un-stimulated cells the negative control. The original area of the collagen gels was the area (2 cm^2^) of a well of a 24 well plate, before release from the sides of the wells. A decrease in gel area correlates with an increase in contractility and an increase in gel area correlates with relaxation or inhibition of contractility. Percentage increase or decrease in contractility was compared to that of the unstimulated or basal contraction. One way ANOVA with Tukey's post-hoc analysis, were used to statistically analyse the data (GraphPad Prism software 5, GraphPad Software, Inc., La Jolla, CA, USA), P values < 0.05 were considered to be statistically significant.

### Immunofluorescence microscopy

Fluorescence microscopy was performed on hUtSMC embedded collagen gels (equilibrated overnight and 30–105 min post release/treatment). Gels were washed in 1× phosphate buffered saline (PBS) and fixed in 1% paraformaldehyde and permeabilised with 1% TritonX-100/1 × PBS. The blocking solution was 1% bovine serum albumin (BSA)/PBS. The primary antibody was a 1/100 dilution of a fluorescein isothiocyanate (FITC) labelled monoclonal mouse anti-human SMα-Actin antibody (Sigma, Ireland) in 1%BSA (wt/vol)/1 × PBS in 500 μl at 4°C overnight. The gels were washed and then incubated in 1 × PBS 1/400 dilution of secondary anti-mouse antibody in 1%BSA (wt/vol)-1 × PBS. Fluorescent images were obtained using the DP70 fluorescence microscope (Olympus, Tokyo, Japan).

### Scanning electron microscopy

Samples (collagen cell lattices, 1–5 hrs post release/treatment, with at least 8 hrs pre-equilibration) were washed in 0.1 M phosphate buffer and then fixed in 2.5% (vol/vol) glutaraldehyde. The gels were then washed in phosphate buffer and dehydrated in 50–100% ethanol. Hexamethyldisilazane was added and samples were air-dried and mounted on scanning electron microscopy stubs using a carbon pad, gold coat. Samples were scanned on an S-4700 scanning electron microscope (Hitachi, Japan).

### RNA extraction

hUtSMC embedded collagen gels were digested with diethyl pyrocarbonate (DEPC) treated collagenase I (Sigma, Ireland) at 37°C for 30 mins and centrifuged at 4°C and subsequently washed and centrifuged at 4°C with 1 × PBS (DEPC treated) and sterile RNase free water. Total RNA was isolated from the cell pellet using the RNeasy mini RNA isolation kit (Qiagen, Crawley, West Sussex, UK) and DNase 1-treated (Invitrogen, USA).

### Reverse transcription

RNA (1 μg) was reverse transcribed into complementary DNA (cDNA) with SuperScript III (Invitrogen, USA). Control RNA samples, in which no reverse transcriptase was added, were included to confirm that no genomic DNA contamination was present.

### Polymerase Chain Reaction (PCR)

1 μl of the 20 μl RT reaction was used as template for PCR, performed with 1.25 U Taq DNA polymerase (Bioline Ltd., Taunton, MA, USA), 0.2 mM dNTPs and 0.2 μM of each primer. cDNA amplification was performed by an initial denaturation step of 5 minutes at 95°C followed by 28–40 cycles of denaturation at 94°C for 1 min, annealing at 55–60°C for 1 min and elongation at 72°C for 30 s–1 min, and a final extension step at 72°C for 10 minutes. The sequences of the oligonucleotide primers (MWG, Ebersberg, Germany) were:

*ACTB *(β-Actin)

5'-CAACTCCATCATGAAGTGTGA-3'

5'-GCCATGCCAATCTCATC-3' (Accession M10277)

*PTGS2 *(Prostaglandin endoperoxide synthase 2-COX-2)

5'-GTGCAACACTTGAGTGGCTAT-3'

5'-AGCAATTTGCCTGGTGAATGAT-3' [19]

*ESR1 *(Estrogen receptor 1)

5'-ACAAGGGAAGTATGGCTATGGA-3'

5'-GGTCTTTTCGTATCCCACCTTTC-3' [19]

*TAGLN *(Transgelin, smooth muscle 22α)

5'-TTGAAGGCAAAGACATGGCAG-3'

5'-CCATCTGAAGGCCAATGACAT-3' [19]

*CNN1 *(Calponin 1)

5'-CGAAGACGAAAGGAAACAAGGT-3'

5'-GCTTGGGGTCGTAGAGGTG-3' [19]

*PGR *(Progesterone receptor)

5'-CAAAACCTGACACCTCCAGTT-3'

5'-GCCACATGGTAAGGCATAATGA-3' [19]

*ACTA2 *(Smooth muscle α actin)

5'-ACAACAGCATCATGAAGTGT-3'

5'-CCAGTAGCCTATTTCAGATT-3' (Refseq NM_001613)

### Real time fluorescence PCR

Real time fluorescence PCR was performed using the Applied Biosystems StepOne Plus™ Real Time PCR System Relative Standard Curve method (ABI, Foster City, CA, USA). Standard curves were created for both a housekeeping gene (*ACTB*) and the gene of interest, using DNA of known concentration as template, with ABI Fast Sybr Green (2×) and Fast Optical 96 well reaction plates (ABI, USA). The concentration of each primer was 0.4 μM and template cDNA was 1/10–1/25 dilutions of cDNA from RNA isolated from collagen embedded and treated hUtSMCs. cDNA amplification was performed by an initial step of 95°C for 20 seconds, followed by 40 cycles of denaturation at 95°C for 3 seconds, annealing at 60°C for 30 seconds. The sequences of the oligonucleotide primers for *ACTA2*, *TAGLN *and *PTGS2 *were as above and the sequences for the additional oligonucleotide primers for real time PCR were:

F2R

5'-CAAATGCCACCTTAGATCCCC-3'

5'-CTTCTGAGATGAATGCAGGAAGT-3' [19]

*ACTB *(β-Actin)

5'-GGGCATGGGTCAGAAGGATT-3'

5'-AGTTGGTGACGATGCCGTG-3' (Accession M10277)

Fluorescence data was acquired at the end of each PCR cycle. Melting curve analysis was performed by an initial denaturation step of 95°C for 15 seconds and 60°C for 1 minute and 95°C for 15 seconds. Fluorescence was measured continually during the melting curve cycle. Each reaction was performed in triplicate. With the relative standard curve method, the StepOne Plus™ Real Time computer software (ABI, USA) measured amplification of *F2R *and the endogenous control, the housekeeping gene *ACTB *in samples, reference sample (unstimulated collagen embedded cells) and in the standard dilution series, all on the same reaction plate. Measurements were normalised using the endogenous control, *ACTB*, where the *F2R *quantity mean sample value is divided by the corresponding *ACTB *value. Data from the standard dilution series were used to generate the standard curve. Using the standard curve, the software interpolated target quantity in the samples and the reference sample (un-stimulated collagen embedded cells). The software determined the relative quantity of target in each sample by comparing target quantity in each sample to target quantity in the reference sample. Mean *ACTB *normalised starting RNA relative quantities ± SEM from treated collagen embedded hUtSMCs were compared to control or unstimulated cells in collagen gels ± SEM and fold changes calculated. Statistical analyses, student t-tests or one way ANOVA with Tukeys post hoc tests were performed using Graphpad Prism 5 software (GraphPad Software, Inc., USA). P values < 0.05 were considered to be statistically significant.

## Results

### Characterisation of collagen embedded hUtSMC and hTERT-HM cells

**Figure 1:**
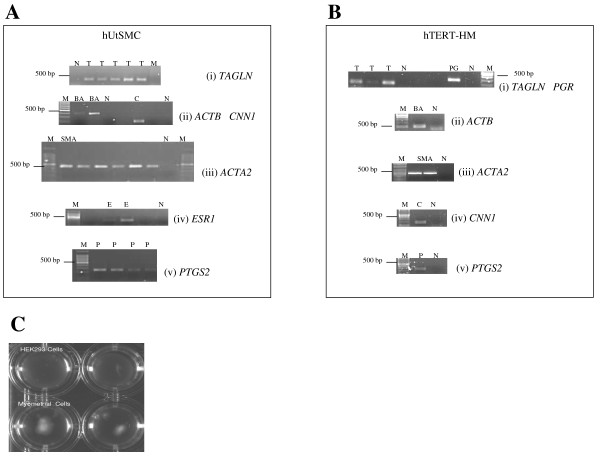
**(A) Representative gel pictures of RT-PCR reactions of (i) *TAGLN *(T) DNA markers (M) PCR water negative (N) are indicated, (ii) *ACTB *(BA) and *CNN1 *(C), DNA markers (M) and PCR negative controls (N) for each gene, (iii) *ACTA2 *(SMA), DNA markers (M), and PCR negative control (N) (iv) *ESR1 *(E), DNA markers (M) and PCR negative control (N) and (v) *PTGS2 *(P), DNA markers (M) on RNA isolated from collagen embedded human uterine smooth muscle cells.(B) **Representative gel pictures of RT-PCR reactions of (i) *TAGLN *(T) and *PGR *(PG), DNA markers (M) and PCR water negatives for both genes (N) respectively, are indicated (ii) *ACTB *(BA) DNA marker (M) and PCR negative (N) (iii) *ACTA2 *(BA) DNA marker (M) and PCR negative (N) (iv) *CNN1*(C) DNA marker and PCR negative (N) (v) *PTGS2 *(P) DNA marker (M) and PCR negative (N), on RNA isolated from hTERT-HM cells. **(C) **A visible decrease in gel area of hUtSMC embedded collagen gel lattices is demonstrated as a result of basal contraction, in comparison to no change in gel area with the collagen embedded HEK293 cells.

The collagen cultured primary hUtSMCs (to passage 8) expressed mRNA for the smooth muscle differentiation markers *TAGLN*, *CNN1 *and *ACTA2*, plus *ESR1 *(Figure 1A(i–iv)). Cultured hUtSMCs also expressed *PTGS2 *(Figure 1A(v)) and *OXTR *(oxytocin receptor) mRNA (data not shown). The hTERT-HM cells expressed *TAGLN*, *PGR*, *ACTA2*, *CNN1 *and *PTGS2 *(Figure 1B(i–v)). The hUtSMC and hTERT-HM cells caused basal contraction of collagen gels whereas the non-contractile HEK293 cells did not (Figure 1C).

### Effect of TNFα treatment on hUtSMC collagen contractility

**Figure 2:**
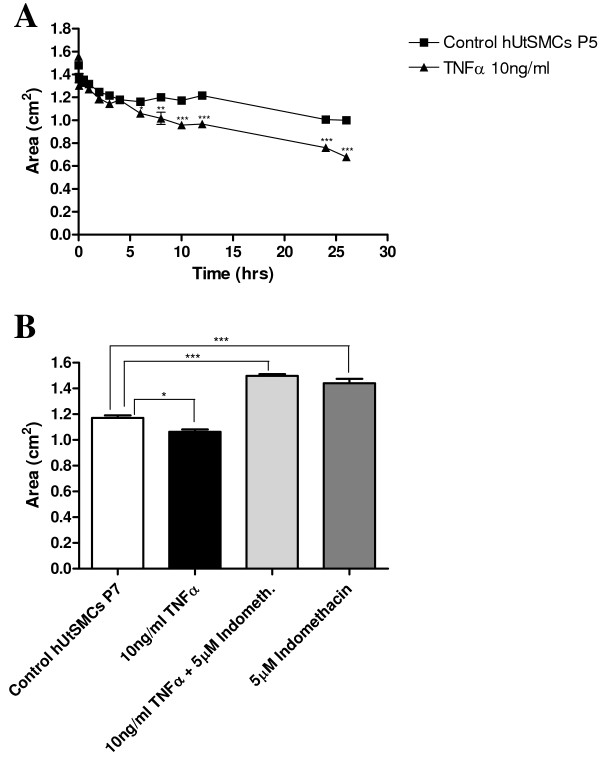
**(A) A time-course experiment of mean gel area (cm^2^) ± SEM (indicated by the error bars) after TNFα (10 ng/ml) treatment of hUtSMC collagen gels**. Significance values are indicated for TNFα treated versus control unstimulated cells, * P < 0.05, ** P < 0.01, *** P < 0.001. **(B) **Mean gel area (cm^2^) ± SEM (indicated by the error bars) after TNFα (10 ng/ml), indomethacin (5 μM), and TNFα/indomethacin treatment of hUtSMC collagen gels 2 hrs 30 mins hours post-treatment. Significance values are indicated for treated cells versus control, * P < 0.05, *** P < 0.001.

TNFα (10 ng/ml) increased collagen contractility, with a decrease in gel area of 9.5% (P < 0.05) to a peak of 47.3% (P < 0.001), 6 to 26 hrs respectively, after treatment, in comparison to un-stimulated collagen embedded hUtSMCs (Figure 2A). Indomethacin (5 μM), a non-selective COX inhibitor, added 30 minutes prior to TNFα treatment, inhibited the TNFα-induced contraction, resulting in an increase in collagen area of 28% (P < 0.001), at 2 hours 30 minutes. Indomethacin (5 μM) alone, inhibited basal contraction; a 23% increase (P < 0.001) in gel area was observed in comparison to control hUtSMC embedded collagen gels, 2.5 hours after its addition (Figure 2B).

### Effect of thrombin treatment on hUtSMC collagen contractility

**Figure 3:**
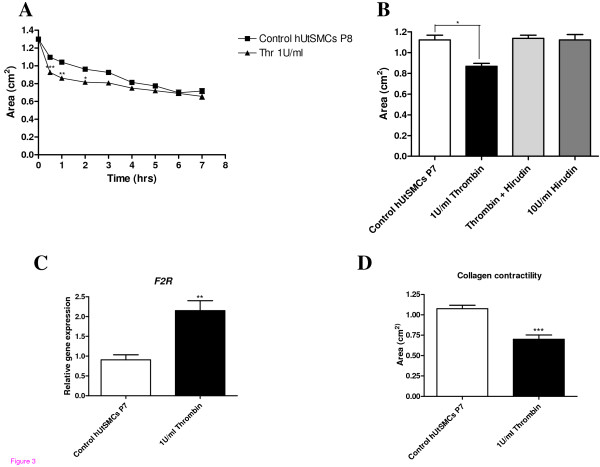
**(A) Mean gel area (cm^2^) ± SEM (indicated by the error bars) of thrombin treatment of hUtSMC embedded gel time-course experiment (0 to 7 hrs)**. Significance values are indicated, * P < 0.05, ** P < 0.01, *** P < 0.001. **(B) **Mean gel area (cm^2^) ± SEM (indicated by the error bars) of thrombin, hirudin and thrombin plus hirudin treatment of hUtSMC embedded gels at 3 hr post-treatment. Significance values are indicated, * P < 0.05. **(C) **Graphical representations of real-time fluorescence RT-PCR results of *ACTB *normalised relative gene expression plotted against control or thrombin treated collagen gel hUtSMC mRNA for *F2R *± SEM (indicated by the error bars) performed in triplicate. **(D) **RNA for the real time fluorescence RT-PCR experiment was isolated from these hUtSMC collagen gels stimulated with 1 U/ml thrombin or control unstimulated gels after 2 hrs. The mean gel area (cm^2^) ± SEM is indicated by the error bars. Significance values are indicated, ** P < 0.01, *** P < 0.001.

Thrombin (1 U/ml) induced a 15.5% increase (P < 0.001) in contractility after 30 minutes which increased to 17.3% at 1 hour (P < 0.01) (Figure 3A). A bar chart representation from a separate experiment illustrated a 29.3% (P < 0.05) decrease in collagen gel area after thrombin application alone, at 1 hr 30 mins. Hirudin (10 U/ml), a thrombin inhibitor, inhibited the thrombin induced contraction, while alone it did not affect basal levels of contractility (Figure 3B).

### Real time RT-PCR analysis of thrombin treated collagen embedded hUtSMC mRNA

Total RNA was isolated from collagen embedded hUtSMCs after a contractility experiment in which cells cultured overnight in collagen in serum free medium were stimulated with 1 U/ml thrombin for 2 hours, resulting in a 35% increase (P < 0.001) in contractility (Figure 3D). RNA was isolated immediately after the area measurement for the 2 hr time-point was acquired.

Relative quantitative expression analysis was performed by real-time fluorescence RT-PCR. In order to minimise any undue experimental error from sources such as pipetting inaccuracies, analysis of each gene was performed in triplicate. The collagen embedded hUtSMCs demonstrated expression of the thrombin receptor *F2R *and the housekeeping gene, *ACTB *mRNA. RT-PCR product specificity was confirmed using melting curve analysis. Amplification curve crossing points were determined for each gene generated within the initial phase of exponential amplification.

The mean quantities per starting mRNA, for the treated and control cells for *F2R *and the housekeeping gene (*ACTB*) were interpolated from the corresponding CT values from the standard curves, using the relative standard curve method StepOne software (ABI, USA). These *ACTB *normalised values, were averaged and values determined for both treated (*n *= 3) and non-treated (*n *= 3). The mean *ACTB *normalised RNA quantities for treated and control ± SEM, for *F2R *were 0.215 ± 0.025, 0.09083 ± 0.01282, this is graphically represented in Figure 3C), with a resultant relative fold increase of 2.36 in the thrombin treated versus the untreated collagen gels.

### Thrombin and Y-27632 modification of hTERT-HM collagen contractility

**Figure 4:**
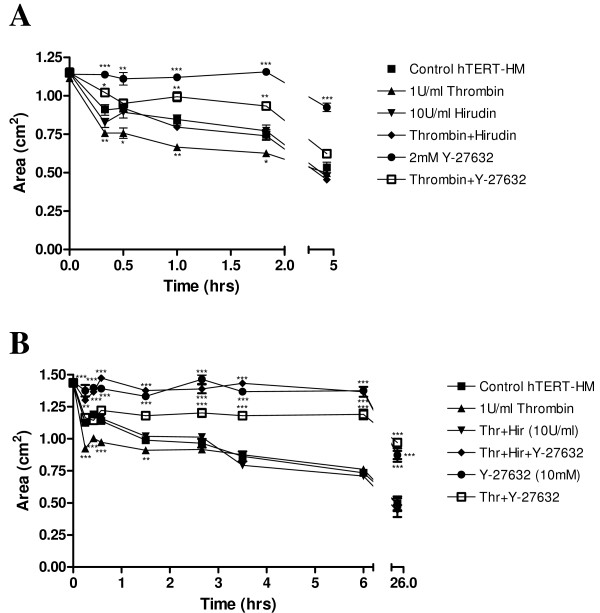
**(A) Time-course experiment of mean gel area (cm^2^) ± SEM (indicated by the error bars) 20 mins to 4 hrs 20 mins after thrombin (1 U/ml), hirudin (10 U/ml), thrombin plus hirudin, Y-27632 (2 μM), thrombin plus Y-27632 or control unstimulated cells in collagen**. Significance values are indicated for the various treatments compared to the control unstimulated collagen embedded cells, * P < 0.05, ** P < 0.01, *** P < 0.001. **(B) **Time-course experiment of mean gel area (cm^2^) ± SEM (indicated by the error bars) 0 to 24 hrs after thrombin (1 U/ml), thrombin plus hirudin (10 U/ml), thrombin plus hirudin plus Y-27632 (10 μM), Y-27632 (10 μM), thrombin plus Y-27632 treatment of hTERT-HM collagen embedded gels versus control unstimulated cells. Significance are indicated for treated cells versus control, ** P < 0.01, *** P < 0.001.

Thrombin (1 U/ml) caused a significant decrease in gel area (Figure 4A and 4B), from 36.3% at 15 mins (P < 0.001) to 17.1% at 35 mins, in comparison to basal contraction of hTERT-HM cells in the collagen gels (Figure 4B). Hirudin (10 U/ml) blocked the thrombin-induced increase, with a return to basal levels of contractility at all time-points analysed (Figure 4A and 4B). Hirudin alone had no significant effect on contractility (Figure 4B). The application of Y-27632 (2 μM) to the hTERT-HM collagen lattices resulted in an inhibition or decrease of basal contraction, with a 24.7% (P < 0.001) at 20 mins, to 73% (P < 0.001) at 4 hr 20 min increase in gel area, in comparison to the untreated collagen hTERT-HM gels. The contractile effect of thrombin was reduced by Y-27632, with a decrease in contractility in the Thr + Y-27632 treated cells compared to those treated with thrombin alone, ranging from 25% at 30 min (P < 0.001) to 49% at 1 hr (P < 0.01) and 1 hr 50 min (P < 0.001), back to 25% at 4 hr 30 min (P < 0.01) (Figure 4A).

The next aim was to analyse the effect of a longer incubation time and a greater concentration of Y-27632 on thrombin induced and basal contractility. The application of Y-27632 (10 μM) resulted in a decrease in contractility (increase in gel area) over basal levels, of 22.2% (P < 0.001) at 15 mins to 75.4% (P < 0.001) at 24 hrs. The contractile effect of thrombin was antagonised by the application of 10 μM Y-27632 with a decrease in contractility in the Thr+Y-27632 treated gels versus those with thrombin treatment alone, ranging from 14% at 25 min (P < 0.001) to 31.8% at 2 hr 40 min (P < 0.001), 56.3% (P < 0.001) to 99% at 24 hrs. On addition of the thrombin inhibitor hirudin, to Thr+Y-27632, contractility levels returned to those of Y-27632 alone (Figure 4B).

### Fluorescence microscopy of collagen embedded hUtSMCs

**Figure 5:**
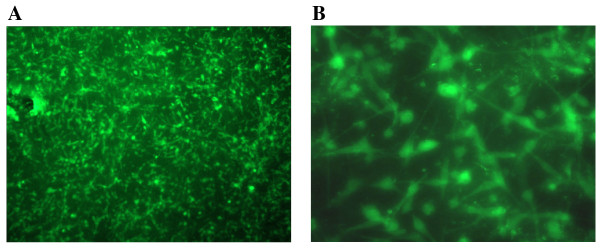
**Fluorescence microscopy of smooth muscle α-actin FITC (green) labelled hUtSMCs in collagen embedded gels**. Original magnification (A) ×4 (B) ×20.

Fluorescence microscopy revealed SMα-Actin FITC immunolabelling of hUtSMCs within the collagen gels in Figure 5(A) and 5(B). It was apparent that the cells demonstrated typical smooth muscle characteristics with an elongated shape and were forming networks.

### Scanning electron microscopic analysis of collagen embedded hUtSMCs

**Figure 6:**
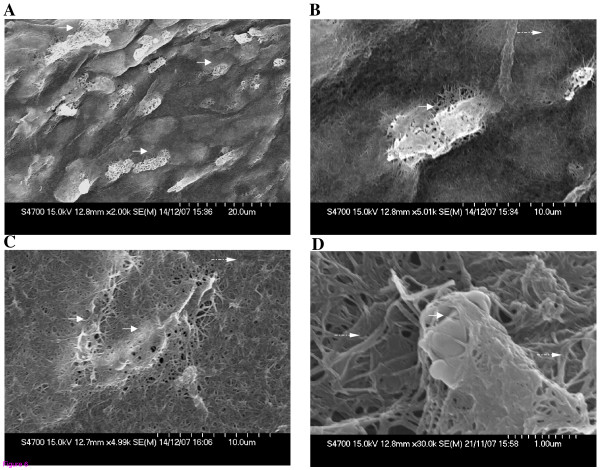
**Scanning electron microscopy of (A-D) hUtSMCs within the collagen gels**. Cells are indicated with continuous arrows and collagen fibrils demonstrated with broken arrows.

Scanning electron microscopic analysis confirmed the incorporation of the hUtSMCs within the 3D structure of collagen fibres. The micrographs demonstrated the cells entrapped within the collagen meshwork, as indicated (Figure 6(A–D). In Figure 6A multiple cells are demonstrated. An individual cell was visible with collagen fibrils entwined about it (Figure 6D).

## Discussion

We studied the effects of a range of uterotonic and uterorelaxant agents on human primary uterine smooth muscle or immortalised human myometrial hTERT-HM cells in an *in vitro *model of human myometrium. In this system the cells were embedded within the collagen gels like that reported by other investigators [5,20] using human myometrial smooth muscle cell lines, and in comparison to another study in which the cells were layered on top of pre-established collagen lattices [7]. The cells were allowed to pre-equilibrate in collagen gels overnight for 12 hours compared to reports in which the myometrial smooth muscle cells were allowed to settle for 2 or 3 days [5,21]. The gel area was measured at intervals, varying from 5 minutes to 24 hours after treatment, whereas in other uterine cell studies, measurements were taken 12–24 hours post-treatment [7]. This ability to modulate contraction minutes after introduction of the various compounds is not dissimilar in certain circumstances to the *in vivo *situation in the myometrium or indeed in the tissue bath setting where responses are quick (in some cases, minutes). As with any *in vitro *model it is not without its limitations, however it will be useful as an initial tool to assess a broad range of potential tocoloytic agents and may be employed as a complementary tool alongside the use of myometrial strips. The ability to monitor gene expression, the ease of use and reproducibility thus make it a suitable model of human myometrial contractility.

Elevated levels of the cytokine TNFα, are found in pregnancies complicated by infection and preterm labour and in normal labour in humans [15,22] and other species [23]. Experimental administration of TNFα can induce both preterm labour and intermediate steps in the labour cascade, such as increased synthesis and decreased degradation of prostaglandins, expression of contraction-associated proteins, and increased uterine contractile activity [24,25]. In agreement with our data TNFα stimulated contractility in uterine smooth muscle and endometrial stromal cell collagen contractility models [20,26]. The non-selective cyclooxygenase (COX) inhibitor indomethacin, clinically used to delay premature labour, inhibited collagen contraction. It reduces uterine contractions through inhibition of prostaglandin synthesis in the uterus, and also possibly through calcium channel blockade [27,28]. The ability of this system to monitor its effect on contractility is extremely beneficial for the testing of future tocolytic agents.

It had previously been established that thrombin enhanced myometrial contractions in human and animal myometrium [10,29,30]. We reported the up-regulation in gene expression of two of its cellular receptors, *F2R *and *F2RL3 *at labour [12]. We have now determined for the first time that thrombin significantly increased both hUtSMC and hTERT-HM collagen contractility and also up-regulated gene expression of its receptor, *F2R*, in this system. Other investigators have described that thrombin also stimulated contraction of human lung and gingival fibroblast cells in other collagen gel systems [31,32]. The ROCK1 pharmacologic inhibitor, Y-27632 decreased both basal and thrombin induced contractility in this *in vitro *human myometrial model, which has not been previously described. This data suggests that the contractile effect of thrombin may in part be mediated by the Rho kinase pathway. Further investigation however is necessary to elucidate the mechanisms involved in thrombin mediated contractility.

The immortalised hTERT-HM cells (which are derived from human myometrium) responded to thrombin in a similar manner to the primary hUtSMC cells and therefore will be a suitable cell line for future use in this *in vitro *human myometrial model. The collagen embedded hTERT-HM cells are responsive to Y-27632, while hirudin and Y-27632 also modified thrombin induced contractility in this system. Furthermore, the ability to passage these cells for a much longer time than primary cells is extremely advantageous.

## Conclusion

This study established that TNFα and thrombin induced contractility in this human myometrial model and indomethacin decreased basal contractility. *F2R *mRNA was up-regulated upon thrombin stimulation of collagen contractility. Y-27632 decreased both basal and thrombin induced contractility. Therefore, it can be concluded that thrombin-induced uterine smooth muscle cell collagen contractility is mediated by F2R activation and may in part be ROCK-1 dependent. The capacity of the gels to consistently contract or relax after treatment with various uterine agents, in a similar timeframe to that in the *in vivo *myometrial situation highlights this as a viable method to evaluate the effects of a wide range of putative myometrial responsive compounds. The ability to isolate total RNA from the collagen cell matrix represents a chance to gain a unique insight into gene expression changes as a result of alterations in collagen contractility. Furthermore, this *in vitro *human myometrial model will enhance our understanding of the many complex biochemical pathways involved in contractility at labour, and possibly contribute to the development of diagnostic technologies and/or therapeutic interventions for the treatment of preterm labour, and other complications of abnormal labour.

## Competing interests

The authors declare that they have no competing interests.

## Authors' contributions

JF performed the hUtSMC collagen contractility studies, SEM, fluorescence microscopy and hUtSMC RNA isolation. JJM provided advice, read and edited the final document. TJS acquired funding, provided advice, read and edited the final document. MOB conceived the idea and design of the project, acquired the data, drafted the manuscript, performed RT-PCRs, real time fluorescence RT-PCR and hTERT-HM collagen contractility studies. All authors read and approved the final document.
